# The effect of ageing on the mechanical properties of the silk of the bridge spider *Larinioides cornutus* (Clerck, 1757)

**DOI:** 10.1038/srep24699

**Published:** 2016-05-09

**Authors:** Emiliano Lepore, Marco Isaia, Stefano Mammola, Nicola Pugno

**Affiliations:** 1 Laboratory of Bio-inspired & Graphene Nanomechanics, Department of Civil, Environmental and Mechanical Engineering, University of Trento, Via Mesiano 77, 38123 Trento, Italy; 2 Laboratory of Ecology and Terrestrial Ecosystems, Department of Life Science and Systems Biology, University of Torino, Via Accademia Albertina, 13, 10123 Torino, Italy; 3 Centre of Materials and Microsystems, Bruno Kessler Foundation, via Santa Croce 77, 38122 Trento, Italy; 4 School of Engineering and Materials Science, Queen Mary University, Mile End Rd, London E1 4NS, UK

## Abstract

Spider silk is regarded as one of the best natural polymer fibers especially in terms of low density, high tensile strength and high elongation until breaking. Since only a few bio-engineering studies have been focused on spider silk ageing, we conducted nano-tensile tests on the vertical naturally spun silk fibers of the bridge spider *Larinioides cornutus* (Clerck, 1757) (Arachnida, Araneae) to evaluate changes in the mechanical properties of the silk (ultimate stress and strain, Young’s modulus, toughness) over time. We studied the natural process of silk ageing at different time intervals from spinning (20 seconds up to one month), comparing silk fibers spun from adult spiders collected in the field. Data were analyzed using Linear Mixed Models. We detected a positive trend versus time for the Young’s modulus, indicating that aged silks are stiffer and possibly less effective in catching prey. Moreover, we observed a negative trend for the ultimate strain versus time, attesting a general decrement of the resistance force. These trends are interpreted as being due to the drying of the silk protein chains and the reorientation among the fibers.

One of the most supreme examples of animal architecture are spiders’ orb webs, which assume a crucial importance for survival, growth and reproduction of a huge number of spiders. One of the main functions of the orb web is to withstand the high stresses needed to absorb the kinetic energy of flying insect prey^1^,^2^,^3^. Since silk is so important for spiders, it has most probably been subjected to strong selective pressures over the 400 million years of spider evolution and can be regarded as one of the keys to the evolutionary success of spiders^4^,^5^. Spider silk is regarded as one of the best natural polymer fibers especially in terms of low density, high tensile strength and high elongation until breaking giving it great toughness. It also compares well compared to the majority of the best performing artificial fibers^3^,^6^,^7^,^8^,^9^,^10^,^11^,^12^,^13^,^14^,^15^,^16^,^17^,^18^,^19^,^20^,^21^,^22^,^23^,^24^,^25^,^26^.

Environmental factors such as temperature^2^,^3^,^7^, humidity^10^,^27^,^28^,^29^, diet^30^,^31^,^32^ and UV radiation^33^ have been proved to affect spider silk mechanical properties in different ways. In particular, ageing causes the cleavage of hydrogen bonds linking silk proteins, the decay of amino acids via emission of ammonia gas from the silk fiber and even oxidation. These are well-documented degradation processes for silkworm silk and, as they also occur in many polymers^33^ they are likely to be present in spider silk^10^ too. This could lead to a transient rearrangement of the silk polymer chains and in general to less compliant silk fibers determining a reduction in the mechanical properties of the silk as recorded during our experiments^10^,^34^.

Moreover, other factors such as reeling methods^29^,^35^,^36^,^37^, environmental conditions during silk spinning^7^,^10^,^27^,^29^,^38^,^39^ and types of silk (e.g. dragline, viscid or egg sac silk)^14^,^22^,^40^ affect silk diameter whereas the strain rate of tensile testing affects the results of the mechanical silk characterization. Therefore, mechanical performances of silk may vary at the inter-individual as well as the intra-individual level^2^,^13^,^20^,^37^,^41^,^42^.

Although spider silk has been intensively studied, few scientific papers have focused on the effects of ageing on mechanical performance^10^,^34^,^43^. In particular, six- and 720-hours ageing of *Argiope trifasciata* spider MA silk fibers^10^, as well as four- and 1-year ageing of different silks and spiders^33^ and 21-days ageing^43^ were analyzed in literature. The bridge spider *Larinioides cornutus* (Clerck, 1757) has been studied looking at its silk biomechanics^44^,^45^,^46^ and at the contribution of diet to its lifetime reproductive success, growth and lifespan^30^,^31^,^32^. So far, no bio-engineering studies have focused on the effect of time (both silk ageing and spider senescence) on the silk of the bridge spider *L. cornutus*.

In this study, we focused on the silk threads of the bridge orb web spider *L. cornutus*. Our data are referred to mixed types of silk, consisting of at least 2 major ampullate (MA), 2 minor ampullate (mA) and abundant aciniform (AF) silk threads. Spiders use MA silk as radial structural silk in orb webs together with capture spiral threads which are able to dissipate the kinetic energy of prey impacts into viscoelastic deformation^1^,^7^. MA silk is also used as a lifeline in case of danger to rapidly descend or escape from predators thanks to its unique properties even under torsion^20^,^34^,^45^,^47^. In general, MA silk shows extremely high strength and toughness in araneomorph spiders. mA silk threads are used for temporary scaffolding during web construction while AF threads are used to wrap and secure freshly captured prey. Orb weaving spiders such as *L. cornutus* generally renew their webs daily by repairing the old broken silk threads or spinning a new one. As a consequence, no orb web silks are normally required to function for longer than ~1 day^33^ even if the bridge line and frames of orbs can be very long lasting. Most technological and future applications of silk, such as super strong cables or clothing or biomedical scaffolds, need to maintain their mechanical performance over time and the degradation mechanism caused by ageing should be avoided^10^,^30^,^33^,^48^. Thus, it is important to understand how the mechanical properties of spider silk are affected by natural ageing.

In this study, we hypothesize that the mechanical properties of the silk of an orb weaver spider varies as function of time (silk ageing). In particular, we conducted nano-tensile tests on vertical naturally spun silk fibers. We measured stress, strain, Young’s modulus and toughness at different time intervals from spinning (20 seconds) up to one month later, coherently with the most exhaustive study in literature^10^.

In order to provide reliable regression coefficients and highlight significant statistical trends relating the dependent variables (mechanical properties) to silk ageing, we specifically designed the study in order to obtain a suitable dataset (both from the quantitative and qualitative point of view) for the application of Linear Mixed Modelling techniques. Such methods allow us to deal with the potentially confusing of individual variability and sampling period, both of which were appropriately taken into account in the design of the statistical methods.

## Results

### Nano-tensile testing

We performed 240 nano-tensile tests of naturally spun silk threads of six adult females of *Larinioides cornutus* over a period of 1 month of natural ageing. Females were collected in two different sampling periods (Summer and Autumn).

**Figure 1:**
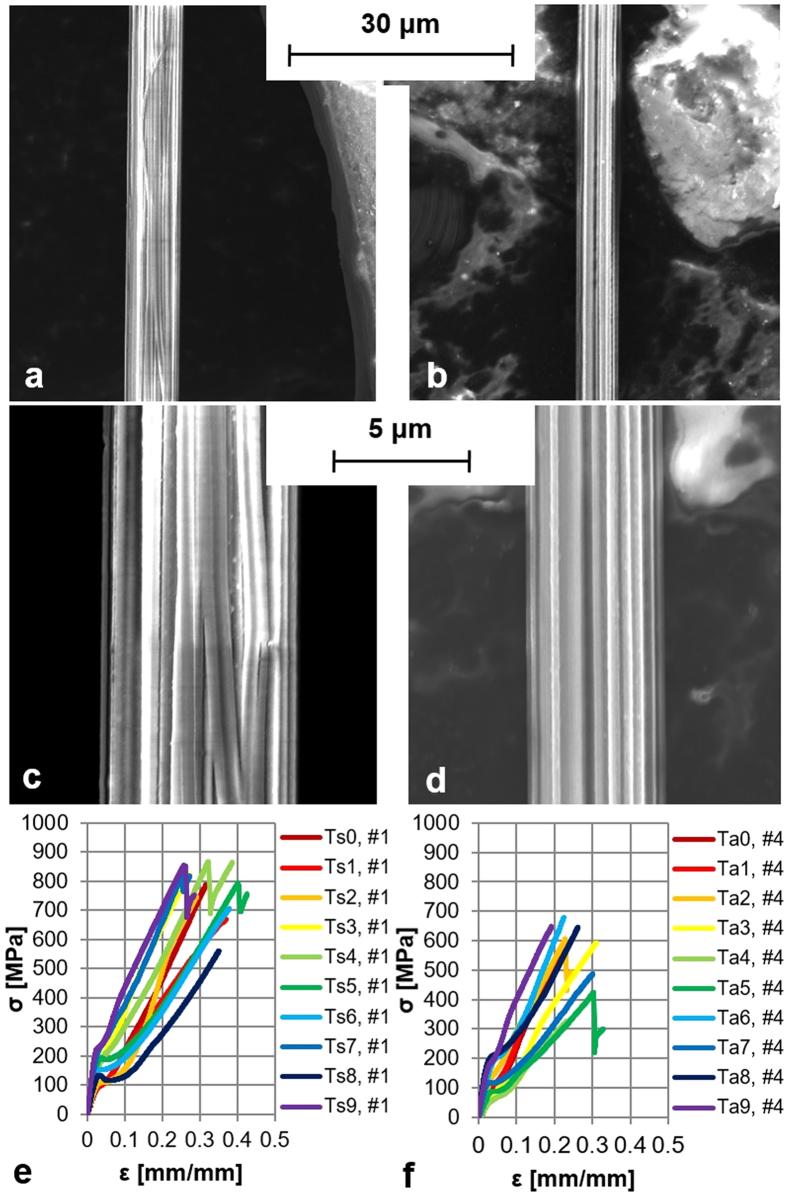
FESEM characterization of silk samples and nano-tensile testing. FESEM micrographies of silk of spider number #2 (**a**) and at high magnification (**c**) and spider number #5 (**b**) and at high magnification (**d**), as representatives of the silk multi-fiber thread diameter of Summer spiders (**a,c**) and Autumn spiders (**b,d**) samples. Stress-strain curves, one at each time interval from spinning, of silk of spider number #1 (**e**) and spider number #4 (**f**), as representatives of stress-strain curves of samples of Summer (**e**) and Autumn (**f**).

**Figure 2:**
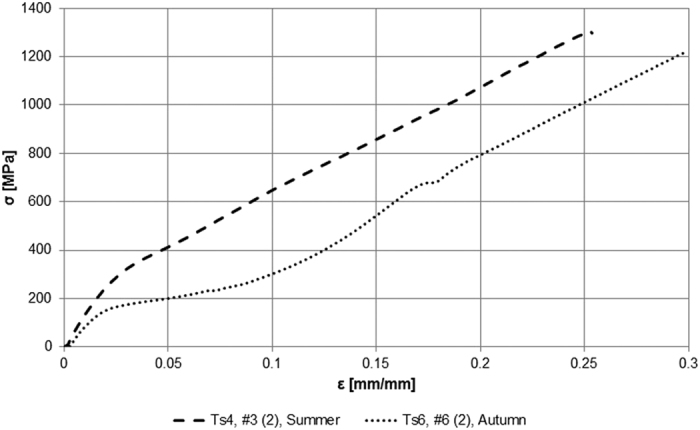
The best performing stress-strain curves of silk threads. Results of the stress-strain curves corresponding to the maximum stress value observed during experiments of Summer (second sample (2) of spider #3) and Autumn silks (second sample (2) of spider #6).

From the various tensile tests (Fig. 1a–f), we obtained rough data of ultimate stress and strain, Young’s modulus and toughness. The maximum failure stress and strain were 1.3 GPa and 117% for Summer silk or 1.1 GPa and 56% for Autumn silk, showing an Autumn strain value higher than the Summer one against the main trend evaluated with LMMs. The maximum value of toughness was 660 MJ/m^3^ for Summer silk and 351 MJ/m^3^ for Autumn silk. Young’s modulus is calculated as the initial slope of the stress-strain curve and the maximum of the Young’s modulus is equal to 14 GPa for Summer silk or 36 GPa for Autumn silk. Figure 2 shows the stress-strain curves corresponding to the maximum values of stress reported above.

### Linear Mixed Models (LMMs)

In order to evaluate the behavior of the mechanical properties of the silk versus ageing (up to 1 month), raw data of ultimate stress and strain, Young’s modulus and toughness were analyzed with Linear Mixed Models (LMMs).

**Figure 3:**
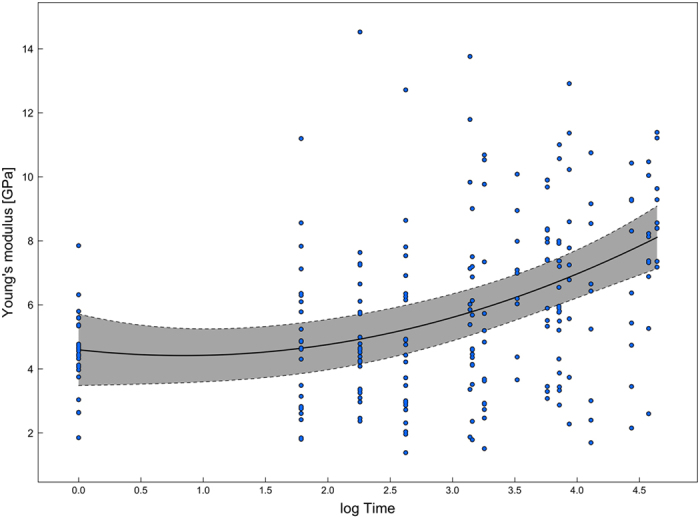
Linear Mixed Models applied to values of Young’s modulus. Observed values of Young’s modulus with a fitted polynomial LMM curve (solid line) and 95% confidence band (grey surface) (details in the Supplementary information).

There was a significant increase in the Young’s modulus over time (log Time^2^ estimates: 0.2557; std. err.: 0.082; p: 0.002; Fig. 3), but no significant variation in respect to sampling period (Autumn estimates: − 0.2859; std. err.: 0.7806; p: 0.7327). The latter variable was therefore dropped during model selection.

**Figure 4:**
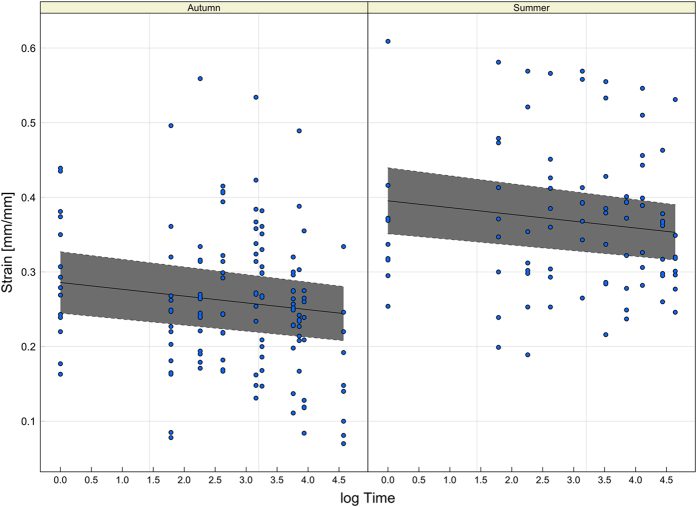
Linear Mixed Models applied to strain values. Observed values of strain with a fitted LMM curve (solid line) and 95% confidence bands (grey surface) in the two sampling periods. The relation versus time is significant for both sampling periods.

There was a significant decrease in ultimate strain values over time (log Time estimates: − 0.009; std. err.: 0.0048; p: 0.051), attesting a general decrease in silk properties with ageing (Fig. 4). We also observed a significant effect of the sampling period, with lower values of strain in Autumn in respect to the reference category Summer (Autumn estimates: − 0.1094; std. err.: 0.023; p: 0.009).

Stress showed no significant decrease over time (log Time estimates: − 12.3709; std. err.: 9.2726; p: 0.183) nor significant difference with the sampling period (Autumn estimates: − 99.6723 ; std err: 44.2764; p: 0.08).

Toughness showed lower values in Autumn in respect to the reference category Summer (Autumn estimates: − 73.2082; std err: 19.0551; p: 0.018), whereas no significant trend over time was observed (log Time estimates: − 0.5919; std. err.: 2.6597; p: 0.824).

Results of model validation are reported in Supplementary Information.

## Discussion

Despite of the fact that our results could be affected by the number and arrangement of fibers composing the tested silk bundle, in general, we obtained stress-strain curves which are coherent with previously tensile test results reported in literature for naturally spun silk threads^2^,^10^,^45^,^49^ and drops of stress values during testing^45^,^50^.

Indeed, we collected multi-stranded silk threads composed of at least 2 major ampullate, 2 minor ampullate and abundant aciniform threads while the spider was descending. As a consequence of the presence of parallel multi-fibers, sudden slight drops of stress values are sometimes exhibited during tensile test before failure (see Fig. 1e,f), but the stress-strain curves suddenly recover to their previous stiffness after these drops^45^. Note that the mechanical properties of a multi-fiber thread exhibiting this behavior were not noticeably different from tests where these drops did not appear and all the silk multi-fibers broke together.

Concerning the variation in the silk properties with ageing, we observed a non-linear increase in the Young’ Modulus values (Fig. 3), as well as a linear decrease of ultimate strain values over time (Fig. 4). On the contrary, ultimate stress and toughness did not vary with time, at least up to 1 month.

More specifically, silk ageing induces an increase of about + 88% in the Young’s Modulus, which is ~6 times higher than the increment of the dragline silk of *Argiope trifasciata*^10^ and ~1.6 or 2.6 times higher than that measured for 1- or 4-year-aged dragline silks of different species of Araneomorphae^33^. In biological terms, aged silks are then stiffer and possibly less effective in catching prey. We also recorded a slight percentage decrement of − 13% in the ultimate stress. These results are not in line with values reported in literature^33^, where values are stable for 1-year-aged dragline silks but − 35% for 4-year-aged dragline silks. Toughness, on the other hand, showed no significant trend in respect to silk ageing, which is in line with results for 1-year-aged dragline silks reported in literature^33^.

Overall, despite the individual variability and other ecological factors such as environment and food intake that may influence silk properties^30^,^31^,^32^, we underline a general decay of the silk over time. The degradation process during the monitored period of 1 month results probably from the loss of water as the result of establishing the thermodynamic equilibrium between the water content in the fiber and that of the atmosphere reducing the elastic mobility of silk proteins^33^,^51^,^52^. In addition, the highly mobile and extensible “nanosprings” of the silk protein chains could be degraded by processes other than mere drying, such as an increased cross-linking and reorientation among them, which indeed determines an increased stiffening of the silk structure with a simultaneous decrease of ultimate strain values^33^.

The potential effects due to individual variability and sampling season were appropriately taken into account by the statistical methods here adopted. In particular, the use of the mixed regression procedure allowed us to include the possible variability at the individual level and provide a more realistic representation of the observed trends. Moreover, in order to avoid bias in the ageing process, all silk samples were stored under controlled laboratory conditions of temperature and humidity and protected from UV light.

An additional source of bias may arise from the effect of the sampling period (“Summer” and “Autumn” populations). Accordingly, the categorical variable Season was introduced in the regression structure as a fixed term (categorical factors made up of less than five levels can not be introduced as random factors^53^), in order to take into account potential variation induced by the sampling period.

The sampling period explained variation in the data for both ultimate strain and toughness. Although differences between the two sampling periods are statistically significant, such variation could possibly be entirely driven by random variation due to the low number of individuals tested for each period. Tentatively, this trend can also be related to the spider senescence (i.e. its age), owing to the effect of the lower intake of food or, more generally, in relation to the natural decline of metabolic processes at the end of the season. The influence of the spider diet on growth, reproduction, survival and silk production is documented in literature^30^,^31^,^32^,^45^, showing that when resources are scarce, spiders spin thinner silk threads, being able to sustain lower applied loads although the maximum tensile stress remains unaffected. Given the small population size for the two sampling periods (n =  3), however, we are unable to fully support this hypothesis from a statistical standpoint. Inferences on spider senescence would need additional observations from different reproductive stages, and a higher population size for the two seasons.

Nano-tensile tests on vertical naturally spun silk fibers of the bridge spider *Larinioides cornutus* revealed changes with silk ageing in some of the mechanical properties here tested (ultimate strain and Young’s modulus).

The potential technological applications of silk threads with properties that change with ageing could be of huge interest in particular for medical applications as such as reabsorbable scaffolds for muscles, nerves, ligaments tissue repairs, as well as absorbable suture materials.

## Methods

We collected three adult females of *Larinioides cornutus* (Araneae, Araneidae) of comparable size in September 2013 (Summer spiders) and in November 2013 (Autumn spiders). Since larger spiders spin proportionately thicker draglines in order to sustain almost two or three times the body weight of the spider itself^45^, we standardized the thread diameter selecting spiders similar in body size. The dry weight of the autumn spiders ranged from 123.0 to 137.6 mg and from 124.7 to 140.1 mg for the summer ones. Table 1 reports the SEM measurements of diameters of the distal part of tibia and metatarsus of the individuals considered in this study. Spiders were collected at the same site, a small canal on the edge of apple orchards in Costigliole Saluzzo, Province of Cuneo, Italy; Geographical coordinates: 44.566 East, 7.493 North). The sampling site was populated by a very abundant population (approximately 20–30 individuals/m^2^), weaving their webs almost in contact and in some cases attached to each other.

Our model species, the bridge spider *L. cornutus*, is a nocturnal synanthropic species that is very abundant near water and lives in aggregations. Under laboratory conditions, females produce up to 15 cocoons over a lifespan of 1.5 years. All developmental stages are present throughout the year, with adult males abundant mainly in September^31^.

According to^54^, *L. cornutus* is one of very few Central European spiders known to be found in “colonies” in which the orb-webs are in direct contact. Individuals of different generations cooperate at least in web-building (i.e. they share the same framework or irregular “web carpet”).

Spiders were immediately put in plastic boxes and transferred in our lab after collection. All samples were collected from freely climbing spiders, following the purpose of previously published papers in order to obtain stronger silk threads and more reproducible results with less variability^2^,^7^,^9^,^47^,^49^,^50^.

Spider silk samples were collected within 1 hour of the spiders being brought into the laboratory. In order to reduce the possible sources of noise in the analysis^2^,^7^,^9^,^47^,^49^,^50^, the silk was collected using a simple standard method as follows: under controlled laboratory conditions, we forced spiders to fall down vertically from a wooden stick, coherently with previously published papers^10^,^33^,^45^,^50^ providing the first source of highly reproducibility of experimental results^47^,^55^. This collecting method allows spiders to produce stronger silk threads and reflects more closely the natural condition^34^,^45^,^47^,^56^. Another viable alternative is collecting silk spun horizontally, which was not used in this paper. This is advantageous compared to artificial forced spinning methods, which are influenced by different parameters such as the spinning speed and the anaesthetization of the spider. It is evident that the silk flow is under the active control of the spider, which can calibrate the tensile behavior of the silk by varying the fiber diameter and microstructure^10^,^45^. The natural vertically spun silk method adopted here standardizes the spinning of the spider (where the spider falls at a constant speed under its own weight) and it avoids anaesthetizing the spider itself, which has been shown to influence the tensile properties of the spider silk^37^,^39^,^57^. Thus, it can be considered a less invasive method as shown by the more reproducible results, of the vertical naturally spun silk (reported for the spider *Argiope trifasciata*^10^,^47^ or the spider *Nephila maculata*^45^) compared to other forced horizontal spinning methods where the spider must be immobilized and forced to spin silk on a collector^10^,^39^,^55^ or to harvesting from webs^9^. No spider were damaged during the manipulation required for this silking method. The height of spider falls was standardized to 20 cm and the collecting region of the thread was fixed at the mid point of the fall (10 cm) in order to decrease the likelihood that the intrinsic variation of spider speed during each descent could interfere with the reproducibility of results. One fall per collected sample, so each spider fell 3 different times to spin 3 different samples. We successfully collected 3 samples for each Summer spider and 5 for each Autumn spider for each nano-tensile test (3 ×  3 +  3 ×  5 =  24 samples for each of the ten nanotensile tests). Altogether, we collected 90 samples of Summer silk and 150 samples of Autumn silk, coherently with previously published papers^10^,^38^,^45^ (see Supplementary Information). The analysis was carried out up to 1 month after the spinning. After one month a rapid and dramatic so-called “degradation” occurs, as documented in^33^. The first tests were performed at Ts0 namely within 20 seconds of spinning. The rest of the samples were left in the laboratory in a closed black box to avoid the exposure to UV radiation under the same controlled ambient conditions adopted for the collecting procedure and nano-tensile tests. Since the ambient conditions such as temperature and humidity during testing may affect the mechanical characteristics of the silk thread^10^,^27^, we monitored the variation of the experimental ambient conditions with a portable datalogger (EL-USB-2, Lascar Electronics) (see Supplementary Information).

### Nano-tensile testing

Nano-tensile tests were planned at 0.16 (Ts0), 60 (Ts1), 180 (Ts2), 420 (Ts3), 1380 (Ts4), 3300 (Ts5), 7140 (Ts6), 12900 (Ts7), 27300 (Ts8), 43740 (Ts9) minutes after spinning for Summer spiders and at 0.16 (Ta0), 60 (Ta1), 180 (Ta2), 420 (Ta3), 1440 (Ta4), 1800 (Ta5), 5760 (Ta6), 7200 (Ta7), 8600 (Ta8), 37440 (Ta9) for Autumn spiders. Samples were pulled at a constant rate of 1%/s until the silk fibers failed. This strain rate was selected because it is coherent with the parameter setting of many previous studies on spider silk mechanics^2^,^3^,^8^,^12^,^20^,^33^,^44^,^50^,^56^,^58^, so maximizing the comparability of results.

With the extreme accuracy needed for nano-tensile tests it was necessary to correctly evaluate the elongation of each sample, the computer program (Nanosuite^®^, Agilent, Santa Clara, USA) automatically recalculated the exact initial gage length (*l*_0_, previously fixed at approx. 20 mm) at the beginning of each test when any pre-load is recorded by the software. This avoids any over- or under-estimation which might lead to a uncorrected determination of elongation. The length of the initial collected fiber was ~30 cm. Thus, load-extension data were transformed into stress-strain curves to normalize data across silk samples of different lengths. This procedure guarantees an extreme accuracy of the initial gage length compared to the less precise “in web” length^44^ or fixed original initial length^2^,^3^,^33^,^34^,^45^ method for calculating stress σ , strain ε , the Young’s modulus *E* and the toughness (see Supplementary Information).

### Linear Mixed Models (LMMs)

The data exploration was carried out following^59^ (see Supplementary Information). Rather than calculating the mean values of the mechanical parameters and their percentage variation as previously reported in literature^33^, we adopted the LMMs analysis to estimate the significance of the trends versus the two variables, namely to understand if and how the mechanical properties of the silk vary with silk ageing. In order to capture potential non-linear trends in the response of dependent variables, we allowed up to second order polynomial for the independent variable.

Prior to model fitting, the independent variable Time was log transformed to achieve homogeneity. The statistic mixed procedure allowed us to deal with violation of independence derived from the fact that silk samples were collected from the same individual and showed high variability. Rather than to test for its direct effect on the dependent variable (i.e. the different mechanical properties), the “individual” level was included in the analysis as random factor in order to account for the variation it introduced in our samples and thus to correctly estimate the regression coefficient. Moreover, we included in the analysis the sampling season, in order to take into account its possible effect on the silk mechanical properties. Being made up of only two levels, it was not possible to include the latter variable as a random component^53^.

The resulting structure of our models was:


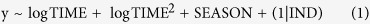


where y =  one of ultimate stress or strain, Young’s modulus and toughness; log TIME =  log transformed time interval from the spinning) (continuous variable, fixed effect), SEASON =  Summer or Autumn (categorical variable, fixed effect). The random part of the model (1|IND) includes the effect of the “individual” variable.

Whenever the effect of a variable was not significant, we simplified the model by dropping it from the initial structure. The process was reiterated until a minimum adequate model of significant fixed effects remained. Model validation was carried out following^60^ (see Supplementary information).

### FESEM characterization of the *Larinioides cornutus* spider silk fibers

The diameter was determined using the FESEM (FEI-InspectTM F50, at 5 kV) micrographs of the silk threads of individual #2 and #5 for Summer and Autumn spiders. Silk threads appears to be composed of parallel multi-fibers of circular cross-sections (at least 2 major ampullate, 2 minor ampullate and abundant aciniform threads), as shown in Fig. 1a–d and confirmed in previously published papers^42^. We made no attempts to isolate single fibers in order to preserve the silk structure the spider naturally spins. For mechanical characterization, the average diameter of silk samples was measured at five points along the length of the fiber using the SEM. Thus, the cross-sectional area was calculated assuming a circular cross-section so treating the multi-stranded structure as a solid cylinder neglecting the air gaps visible in Fig. 1a–d. This hypothesis was made on the basis of evidence used in two previously published papers with the same scope (for details see^3^) and due to the experimental limitations in isolating single fibers even if this adds uncertainty to the correct assessment of the bundle cross-sectional areas which then leads to a high standard deviation on stress values derived from them. The mean diameter is of ~7.15 ±  0.07 μm (or 5.21 ±  0.05 μm) for Summer (or Autumn) silks.

## Additional Information

**How to cite this article**: Lepore, E. *et al.* The effect of ageing on the mechanical properties of the silk of the bridge spider *Larinioides cornutus* (Clerck, 1757). *Sci. Rep.* **6**, 24699; doi: 10.1038/srep24699 (2016).

## Supplementary Material

Supplementary Information

## Figures and Tables

**Table 1:** SEM measurements of diameters of the distal part of tibia and metatarsus of the individuals considered in this study.

Spider ID:	#1	#2	#3	#4	#5	#6
Distal diameter of tibia [mm]	0.85	0.82	0.74	0.80	0.73	0.84
Distal diameter of metatarsus [mm]	0.33	0.35	0.32	0.36	0.36	0.37

## References

[b1] Vollrath F. Biology of spider silk. Int. J. Biol. Macrmol. 24, 81–88 (1999). 10.1016/s0141-8130(98)00076-2 10342751

[b2] Vollrath F., Madsen B. & Shao Z. The effect of spinning conditions on the mechanics of a spider’s dragline silk. Proc. R. Soc. Lond. B 268, 2339–2346 (2001). 10.1098/rspb.2001.1590 PMC1088885 11703874

[b3] Moore A. M. F. & Tran K. Material properties of cobweb silk from the black widow spider *Latrodectus hesperus*. Int. J. Biol. Macromol. 24, 277–282 (1999). 10342775 10.1016/s0141-8130(98)00090-7

[b4] Craig C. L. Spiderwebs and silk: Tracing evolution from molecules to genes to phenotypes (Oxford University Press, New York, 2003).

[b5] Sensenig A., Agnarsson I. & Blackledge T. A. Behavioral and biomaterial coevolution in spider orb webs. J. Evolution. Biol. 23, 1839–1856 (2010). 10.1111/j.1420-9101.2010.02048.x 20629854

[b6] Lepore E., Marchioro A., Isaia M., Buehler M. & Pugno N. Evidence of the most stretchable egg sac silk stalk, of the European spider of the year *Meta Menardi*. Plos one 7, 1–12 (2012). 10.1371/journal.pone.0030500 PMC3275603 22347380

[b7] Foelix R. F. Biology of Spiders (Oxford University Press, 1996).

[b8] Blackledge T. A. & Hayashi C. Y. Silken toolkits: biomechanics of silk fibers spun by the orb web spider *Argiope argentata* (Fabricius 1775). J. Exp. Biol. 209, 2452–2461 (2006). 16788028 10.1242/jeb.02275

[b9] Perez-Rigueiro J., Elices M., Llorca J. & Viney C. Tensile properties of *Argiope trifasciata* drag line silk obtained from the spider’s web. J. Appl. Polym. Sci. 82, 2245–2251 (2001).

[b10] Elices M., Pérez-Rigueiro J., Plaza G. R. & Guinea G. V. Finding inspiration in *Argiope trifasciata* spider silk fibers. J. Mineral. Metals. Materials Soc. 57, 60–66 (2005).

[b11] Poza P., Perez-Rigueiro J., Elices M. & Llorca J. Fractographic analysis of silkworm and spider silk. Eng. Fract. Mech. 69, 1035–1048 (2002).

[b12] Hayashi C. Y., Blackledge T. A. & Lewis R. V. Molecular and mechanical characterization of aciniform silk: uniformity of iterated sequence modules in a novel member of the spider silk fibroin gene family. Mol. Biol. Evol. 21, 1950–1959 (2004). 15240839 10.1093/molbev/msh204

[b13] Madsen B., Shao Z. Z. & Vollrath F. Variability in the mechanical properties of spider silks on three levels: interspecific, intraspecific and intraindividual. Int. J. Biol. Macromol. 24, 301–306 (1999). 10342779 10.1016/s0141-8130(98)00094-4

[b14] Van Nimmen E., Gellynck K., Gheysens T., Van Langenhove L. & Mertens J. Modelling of the Stress-Strain behaviour of egg sac silk of the spider *Araneus diadematus*. J. Arachnol. 33, 629–639 (2005).

[b15] Gosline J. M., Guerette P. A., Ortlepp C. S. & Savage K. N. The mechanical design of spider silks: from fibroin sequence to mechanical function. J. Exp. Biol. 202, 3295–3303 (1999). 10562512 10.1242/jeb.202.23.3295

[b16] Köhler T. & Vollrath F. Thread biomechanics in the two orb weaving spiders *Araneus diadematus* (Araneae, Araneidae) and *Uloborus walckenaerius* (Araneae, Uloboridae). J. Exp. Zool. 271, 1–17 (1995).

[b17] Römer L. & Scheibel T. The elaborate structure of spider silk. Prion 4, 154–161 (2008). 19221522 10.4161/pri.2.4.7490 PMC2658765

[b18] Gosline J. M., Demont E. M. & Denny M. W. The structure and properties of spider silk. Endeavor 10, 37–44 (1986).

[b19] Ortlepp C. & Gosline J. M. The scaling of safety factor in spider draglines. J. Exp. Biol. 211, 2832–2840 (2008). 18723542 10.1242/jeb.014191

[b20] Swanson B. O., Blackledge T. A., Beltran J. & Hayashi C. Y. Variation in the material properties of spider dragline silk across species. Appl. Phys. A-Mater. 82, 213–218 (2006).

[b21] Zhao A. C. *et al.* Novel molecular and mechanical properties of egg case silk from wasp spider, Argiope bruennichi. Biochemistry 45, 3348–3356 (2006). 16519529 10.1021/bi052414g

[b22] Stauffer S. L., Coguill S. L. & Lewis R. V. Comparison of physical properties of three silks from *Nephila clavipes* and *Araneus gemmoides*. J. Arachnol. 22, 5–11 (1994).

[b23] Denny M. The physical properties of spider’s silk and their role in the design of orb-webs. J. Exp. Biol. 65, 483–506 (1976).

[b24] Dunaway D. L., Thiel B. L. & Viney C. Tensile mechanical property evaluation of natural and epoxide-treated silk fibers. J. Appl. Polym. Sci. 58, 675–683 (1995).

[b25] Cunniff P. M. *et al.* Mechanical and thermal properties of dragline silk from the spider *Nephila clavipes*. Polym. Advan. Technol. 5, 401–410 (1994).

[b26] Agnarsson I., Kuntner M. & Blackledge T. A. Bioprospecting finds the toughest biological material: extraordinary silk from a giant riverine orb spider. Plos one 5, 1–8 (2010). 10.1371/journal.pone.0011234 PMC2939878 20856804

[b27] Agnarsson I., Dhinojwala A., Sahni V. & Blackledge T. A. Spider silk as a novel high performance biomimetic muscle driven by humidity. J. Exp. Biol. 212, 1990–1994 (2009). 19525423 10.1242/jeb.028282

[b28] Brown C. P. *et al.* The critical role of water in spider silk and its consequence for protein mechanics. Nanoscale 3, 3805 (2011). 21837334 10.1039/c1nr10502g

[b29] Liu Y., Shao Z. & Vollrath F. Relationships between supercontraction and mechanical properties of spider silk. Nat. mater. 12, 901–905 (2005). 16299506 10.1038/nmat1534

[b30] Kleinteich A., Wilder S. M. & Schneider J. M. Contributions of juvenile and adult diet to the lifetime reproductive success and lifespan of a spider. Oikos 124, 130–138 (2015).

[b31] Kleinteich A. & Schneider J. M. Developmental strategies in an invasive spider: constraints and plasticity. Ecol. Entomol. 36, 82–93 (2011).

[b32] Kleinteich A. & Schneider J. M. Evidence for Rensch’s rule in an orb-web spider with moderate sexual size dimorphism. Evol. Ecol. Res. 12, 667–683 (2010).

[b33] Agnarsson I., Boutry C. & Blackledge T. A. Spider silk aging: initial improvement in a high performance material followed by slow degradation. J. Exp. Zool. 309, 494–504 (2008). 10.1002/jez.480 18626974

[b34] Blackledge T. A. *et al.* How super is supercontraction? Persistent versus cyclic responses to humidity in spider dragline silk. J. Exp. Biol. 212, 1981–1989 (2009). 19525422 10.1242/jeb.028944

[b35] Boutry C., Řezáč M. & Blackledge T. A. Plasticity in major ampullate silk production in relation to spider phylogeny and ecology. Plos one 6, 1–8 (2011). 10.1371/journal.pone.0022467 PMC3144891 21818328

[b36] Heim M., Keerl D. & Scheibel T. Spider silk: from soluble protein to extraordinary fiber. Angew. Chem. Int. Ed. 48, 3584–3596 (2009). 10.1002/anie.200803341 19212993

[b37] Pérez-Rigueiro J., Elices M., Plaza G., Real J. I. & Guinea G. V. The effect of spinning forces on spider silk properties. J. Exp. Biol. 208, 2633–2639 (2005). 16000533 10.1242/jeb.01701

[b38] Guinea G. V., Elices M., Perez-Rigueiro J. & Plaza G. Self-tightening of spider silk fibers induced by moisture. Polymer 44, 5785–5788 (2003).

[b39] Work R. W. A comparative study of the supercontraction of major ampullate silk fibers of orb-web-building spiders (Araneae). J. Arachnol. 9, 299–308 (1981).

[b40] Van Nimmen E., Gellynck K. & Van Langenhove L. The tensile behaviour of spider silk. Autex Research Journal 5, 120–126 (2005).

[b41] Madsen B., Shao Z. Z. & Vollrath F. Variability in the mechanical properties of spider silks on three levels: interspecific, intraspecific and intraindividual. Int. J. Biol. Macromol. 24, 301–306 (1999). 10342779 10.1016/s0141-8130(98)00094-4

[b42] Silva L. P. & Rech E. L. Unravelling the biodiversity of nanoscale signatures of spider silk fibres. Nat. commun. 4, 3014 (2013). 24345771 10.1038/ncomms4014

[b43] Griffiths J. R. & Salanitri V. R. The strength of spider silk. J. Mater. Sci. 15, 491–496 (1980).

[b44] Swanson B. O., Blackledge T. A. & Hayashi C. Y. Spider capture silk: performance implications of variation in an exceptional biomaterial. J. Exp. Zool. 307, 654–666 (2007). 10.1002/jez.420 17853401

[b45] Heiling A. M. & Herberstein M. E. Activity patterns in different developmental stages and sexes of *Larinioides sclopetarius* (Clerck) (Araneae, Araneidae). Selden P. A. (ed.). Proceedings of the 17th European Colloquium of Arachnology, Edinburgh (1998).

[b46] Wolff J. O., Schneider J. M. & Gorb S. N. How to pass the gap – functional morphology and biomechanics of spider bridging threads. Biotechnology of Silk 5, 165–177 (2014).

[b47] Garrido M. A., Elices M., Viney C. & Perez-Rigueiro J. Active control of spider silk strength: comparison of drag line spun on vertical and horizontal surfaces. Polymer 43, 1537–1540 (2002).

[b48] Lu Q. *et al.* Degradation mechanism and control of silk fibroin. Biomacromolecules 4, 1080–1086 (2011). 21361368 10.1021/bm101422j PMC3404841

[b49] Zemlin L. C. A study of the mechanical behaviour of spider silk. Cloting and organic materials laboratory, US Army Natick Laboratories (1968).

[b50] Blackledge T. A., Swindeman J. E. & Hayashi C. Y. Quasistatic and continuous dynamic characterization of the mechanical properties of silk from the cobweb of the black widow spider *Latrodectus Hesperus*. J. Exp. Biol. 208, 1937–1949 (2005). 15879074 10.1242/jeb.01597

[b51] Hayashi C. Y. & Lewis R. V. Evidence from flagelliform silk cDNA for the structural basis of elasticity and modular nature of spider silks. J. Mol. Biol. 275, 773–784 (1998). 9480768 10.1006/jmbi.1997.1478

[b52] Becker N. *et al.* Molecular nanosprings in spider capture-silk threads. Nat. Mater. 2, 278–283 (2003). 12690403 10.1038/nmat858

[b53] Gelman A. & Hill J. Data analysis using regression and multi level/hierarchical models. New York: Cambridge University Press (2007).

[b54] Marcus S. *Larinioides sclopetarius*, eine parasoziale Spinne Mitteleuropas? Arachnologische Mitteilungen 27/28, 5 (2004).

[b55] Guinea G. V., Elices M., Real J. I., Gutierrez S. & Perez-Rigueiro J. Reproducibility of the tensile properties of spider (*Argiope trifasciata*) silk obtained by forced silking. J. Exp. Zool. 303, 37–44 (2005). 10.1002/jez.a.111 15612009

[b56] Boutry C. & Blackledge T. A. Biomechanical variation of silk links spinning plasticity to spider web function. Zoology 112, 451–460 (2009). 19720511 10.1016/j.zool.2009.03.003

[b57] Madsen B. & Vollrath F. Mechanics and morphology of silk drawn from anesthetized spiders. Naturwissenschaften 87, 148–153 (2000). 10798202 10.1007/s001140050694

[b58] Lawrence B. A., Vierra C. A. & Moore A. M. F. Molecular and mechanical properties of major ampullate silk of the black widow spider, Latrodectus hesperus. Biomacromolecules 5, 689–695 (2004). 15132648 10.1021/bm0342640

[b59] Zuur A. F., Ieno E. N. & Elphick S. C. A protocol for data exploration to avoid common statistical problem. Methods in Ecology and Evolution 1, 3–14 (2010).

[b60] Zuur A. F., Ieno E. N., Walker N. J., Saveliev A. A. & Smith G. M. Mixed effect models and extensions in ecology with R (Springer, New York, 2009).

