# Supplementary material for: The effect of ageing on the mechanical properties of the silk of the bridge spider Larinioides cornutus (Clerck, 1757)
Source: Sci Rep. 2016 May 9;6:24699. doi: 10.1038/srep24699 (PMC4860589; doi:10.1038/srep24699)
Supplement: Supplementary Information [file srep24699-s1.pdf]

Supplementary Information for

The effect of ageing on the mechanical properties of the silk of the orb web spider *Larinioides cornutus* (Clerck, 1757)

Emiliano Lepore[1], Marco Isaia[2], Stefano Mammola[2], Nicola Pugno[1,3,4,*]

[1] Laboratory of Bio-inspired & Graphene Nanomechanics, Department of Civil, Environmental and Mechanical Engineering University of Trento, Via Mesiano 77, 38123 Trento, Italy.

[2] Laboratory of Ecology and Terrestrial Ecosystems, Department of Life Science and Systems Biology, University of Torino, Torino, Italy.

[3] Centre of Materials and Microsystems, Bruno Kessler Foundation, via Santa Croce 77, 38122 Trento, Italy.

[4] School of Engineering and Materials Science, Queen Mary University, Mile End Rd, London E1 4NS, UK.

[*]Corresponding author: nicola.pugno@unitn.it

Supplementary Note 1. The bridge spider *Larinioides cornutus* .

The model species is a very common and easily available Palearctic orb web weaver, occurring in urbanized habitats, especially near water. It is easy to transport in laboratory and its ecology and behavior are extensively documented in literature[1-9].

Supplementary Note 2. Nano-tensile tests.

The engineering stress σ and strain ε, as well as the Young's modulus *E*, were calculated using the following equations (1, 2, 3):

$$\sigma = \frac{F}{A_b} \tag{1},$$

$$\varepsilon = \frac{\Delta l}{l_0} \tag{2},$$

$$E = \left.\frac{\mathrm{d}\sigma}{\mathrm{d}\varepsilon}\right|_0 \tag{3},$$

where *F* is the force measured by the testing machine, A_b is the initial cross-sectional area of the thread and Δl is the change in thread length during test. Toughness was determined for each thread by numerically integrating the stress values over the full range of strain. The spider silk dissipates energy in the volume suggesting intrinsic huge toughening mechanisms.

Supplementary Note 3. Experimental conditions. The laboratory air temperature and the relative humidity across the whole month of experiments were recorded to be 23 ± 1°C and 59 ± 5% for the Summer spiders and 24 ± 2°C and 52 ± 7% for Autumn spiders. Temperature and relative humidity (RH) control was achieved with an accuracy of ± 0.5°C and ± 3% RH. The natural ageing is coherent with previously published papers and useful to understand correctly the performance and causes of silk degradation.

Note that: no specific permits were required for the described field studies, no specific permissions were required for these locations/activities, the location is not privately-owned, and the field studies did not involve endangered or protected species.

Supplementary Note 4. Laboratory experiments. We fixed the silk threads ends to 30 x 30 mm^2 "C" shaped double adhesive cardboard holders, which have a 20 mm gap between arms of the "C" shape. This method allows the thread to be suspended, maintained at its original tension thanks to the presence of the double adhesive tape, and mounted on the testing machine without being damaged.

The tensile tests were conducted using a nano-tensile testing machine (T150, Agilent, Santa Clara, USA), equipped with 500 mN maximum cell load. The T150 is capable of generating load-extension data from very thin fibers with a load resolution of 50 nN and a displacement resolution of less than 0.1 nm. The cardboard holders were placed between the clamps. Once the holders were in place, the clamps were closed and then the sides of the holders were cut, leaving the thread loose between the clamps.

Supplementary Note 5. Linear Mixed Models We log-transformed (logX + 1) the time intervals from spinning to achieve homogeneity and we removed five extreme values (outliers highlighted in Fig. S1) from the mechanical variables (ultimate stress and strain, Young's modulus and toughness). Such variables were related to the continuous log-transformed time variable (logTime) via Linear Mixed Models (LMMs[10]) in R environment[11]. The categorical variable Season, representing the two time period in which the spiders were collected, was also included in the regression structure to take into account for potential variation it introduced in the models.

The regression models were fitted using *nlme* R package[12], selecting Summer as the reference category. The outcome of the models consisted of regression coefficients for Autumn and for silk ageing (logTime). For the categorical variable "Season", the coefficients express the result of pairwise comparisons with the reference category (Summer) whose significance was assessed via Wald tests[13]. Model validation was carried out following[1], plotting the residuals against the fitted values (Figs. S2,S3a), the log Time (Figs. S2,S3b) and the random factor (Figs. S2,S3c), evaluating the normality of the residuals (Figs. S2,S3d) and checking for non-linear patterns in the residuals. In the case of the Young's Modulus, a weak non-linear pattern was indeed detected and modelled via polynomial LMM (i.e introducing in the model the term logT [11]).

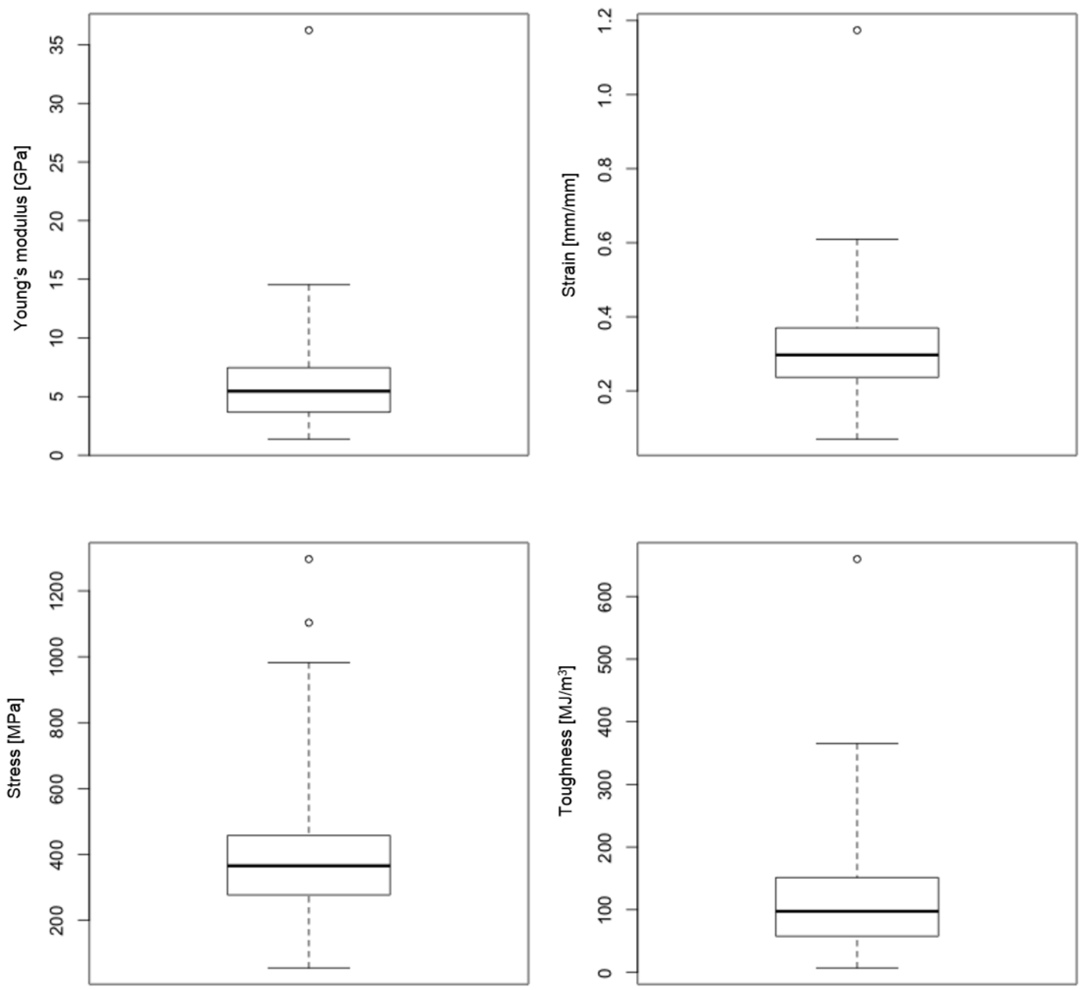

Fig. S1. Boxplots of the dependent variables included in the LMMs. For each variable we represent the medians (bold line), the inter-quartile ranges (25-75%; box), the non-outlier range (dotted line) and the extreme values (above/below the 3.0 inter-quantile ranges; empty circles).

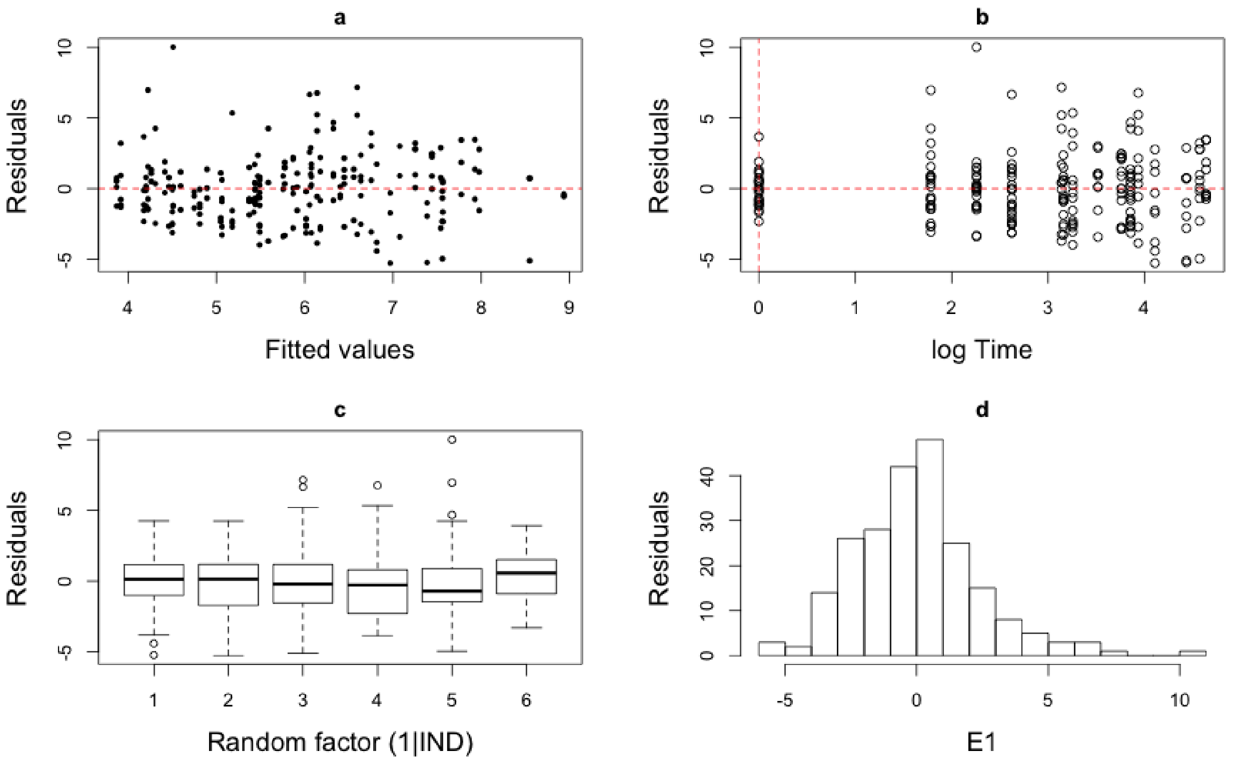

Fig. S2. Standard model validation for Young's modulus. a) Residuals versus fitted values (residual are equally distributed); b) Residuals versus logTime (no patterns are detected); c) residuals versus the random factor (homogenously distributed); d) normality of residuals.

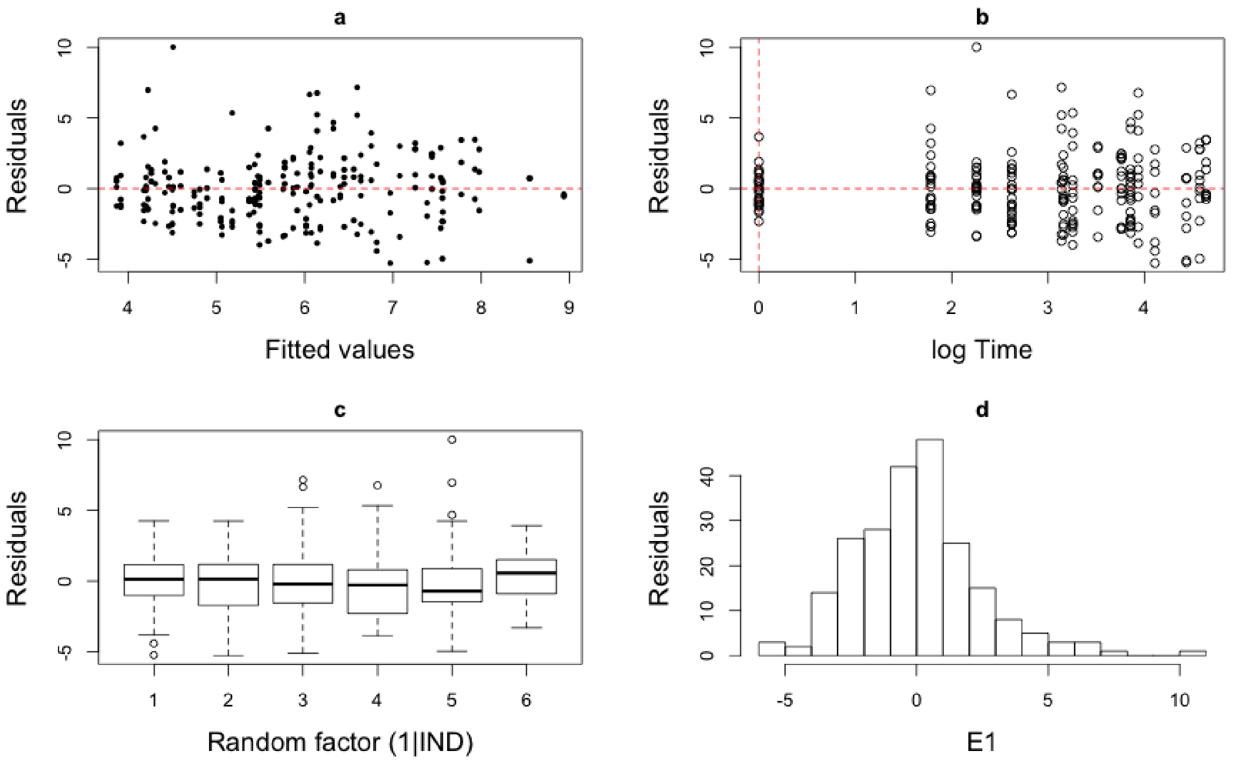

Fig. S3. Standard model validation for Strain. a) Residuals versus fitted values (residual are equally distributed); b) Residuals versus logTime (no patterns are detected); c) residuals versus categorical covariate "Season" (homogenously distributed); d) normality of residuals.

REFERENCES

1. Heiling, A. M. Why do nocturnal orb-web spiders (Araneidae) search for light? *Behav. Ecol. Sociobiol.* **46**, 43-49 (1999).

2. Heiling, A. M. & Herberstein, M. E. The web of Nuctenea sclopetaria (Araneae, Araneidae): relationship between body size and web design. *J. Arachnol.* **26**, 91-96 (1998).

3. Schmitt, M. & Nioduschewski, A. A contribution towards the phenology of Larinioides sclopetarius (Clerck, 1757) (Araneae:Araneidae). Arachnologische Mitteilungen **34**, 9-15 (2007).

4. Schmitt, M. & Nioduschewski, A. Notes on feeding ecology of an urban population of the bridge spider (*Larinioides sclopetarius*) in Duisburg, North Rhine-Westphalia. *Acta Biol. Benrodis* **14**, 89-96 (2007).

5. Kleinteich, A. Life history of the bridge spider, Larinioides sclopetarius (Clerck, 1757). PhD thesis, University of Hamburg, Hamburg, Germany (2010).

6. Schmitt, M. *Larinioides sclopetarius*, a parasocial spider of Central Europe? Arachnologische Mitteilungen **27/28**, 55-67 (2004).

7. Burgess, J. W. & Uetz, G. W. Social spacing strategies in spiders. Spider Communication: Mechanisms and Ecological Significance (ed. by P. N. Witt and J. S. Rovner), 317-351 (Princeton University Press, Princeton, New Jersey, 1982).

8. Kleinteich, A. & Schneider, J. M. Developmental strategies in an invasive spider: constraints and plasticity. Ecol. Entomol. **36**, 82-93 (2011).

9. Kleinteich, A., Wilder, S. M. & Schneider, J. M. Contributions of juvenile and adult diet to the lifetime reproductive success and lifespan of a spider. *Oikos* **124**, 130-138 (2015).

10. Zuur, A. F., Ieno, E. N., Walker, N. J., Saveliev, A. A. & Smith, G. M. *Mixed effect models and extensions in ecology with R* (Springer, New York, 2009).

11. R Development Core Team. *R: a language and environment for statistical computing* (R Foundation For Statistical Computing, 2012).

12. Pinheiro, J., Bates, D., DebRoy, S. & Sarkar, D. *R Development Core Team. nlme: Linear and Nolinear Mized Effect Models* (R package version 3.1-113, Vienna, Austria, 2013).

13. Dobson, A. J. *An introduction to generalized linear models* (Chapman and Hall, New York, 1990).
